# Prospective applications of artificial intelligence for the diagnosis of oral leukoplakia: a scoping review

**DOI:** 10.3389/froh.2026.1760177

**Published:** 2026-02-16

**Authors:** Constanza Jiménez, Carolina Ledesma, Tamara Naranjo, Alejandra Fernández, René Martínez-Flores, Sven Eric Niklander

**Affiliations:** 1 Unit of Oral Pathology and Medicine, Faculty of Dentistry, Universidad Andres Bello, Viña del Mar, Chile; 2 Dermoral Research Group, Laboratory of Translational Dentistry, Faculty of Dentistry, Universidad Andres Bello, Santiago, Chile

**Keywords:** artificial intelligence, diagnosis, deep learning, leukoplakia, oral medicine, machine learning, mouth neoplasms

## Abstract

**Introduction:**

Oral leukoplakia (OL) is the most prevalent oral potentially malignant disorder worldwide. Its diagnosis is clinical and based on excluding all other white patches of the oral cavity, which can be challenging and time-consuming. In recent years, artificial intelligence (AI) has emerged as a promising tool to overcome these limitations, yet a comprehensive overview of the existing evidence is still lacking.

**Objective:**

This scoping review surveys the current landscape of artificial intelligence applications for diagnosing oral leukoplakia, both clinically and histopathologically.

**Materials and methods:**

A comprehensive search was conducted in PubMed, Scopus, Web of Science, and OVID for studies on the use of artificial intelligence for the diagnosis of oral leukoplakia. No date/language restrictions were applied. Two reviewers screened articles and extracted data into predefined tables.

**Results:**

Ten studies were included. Early research used spectroscopy-based models, while recent work employed deep learning for clinical and histopathological image analysis. Most models achieved moderate-to-high diagnostic performance, with sensitivity, specificity and accuracy values above 80%. Overall, models allowed differentiating oral leukoplakia from normal oral mucosa, oral squamous cell carcinoma, and proliferative verrucous leukoplakia, with stronger performance in advanced lesions. Furthermore, artificial intelligence showed promise in grading oral epithelial dysplasia severity in histological samples, occasionally outperforming oral pathologists.

**Conclusions:**

While current evidence remains preliminary, artificial intelligence shows promise as an adjunct tool for oral leukoplakia diagnosis. However, standardized reporting, inclusion of lesions within datasets, and multicenter validation in large and diverse cohorts are still needed to ensure generalizability and further clinical validation.

## Introduction

Oral leukoplakia (OL) is the most common oral potentially malignant disorder of the oral cavity, demanding reliable diagnosis and risk stratification (1). It usually presents as a white plaque that cannot be scraped off and cannot be attributed to any other definable disease (1). OL affects approximately 2.6% of the population (2) predominantly men over 50 years— with a malignant transformation rate estimated in 9.5% (3).

Clinically, OL is classified as homogeneous or non-homogeneous. Homogeneous lesions appear as uniformly white plaques, with smooth, regular surfaces and sharp margins. In contrast, non-homogeneous OLs display mixed white/red patterns, and are further divided into speckled (alternating white and red areas), nodular (with small, polypoid exophytic nodules), or verrucous (characterized by a wrinkled or corrugated surface) (4). Regardless of the subtype, lesion size may range from well-defined millimeter-sized plaques to extensive patches covering broad areas of the oral cavity (4). Non-homogenous lesions are associated with higher degrees of dysplasia and with a higher risk of transforming into an oral squamous cell carcinoma (OSCC) (5, 6).

The diagnosis of OL is clinical, often mimicking other white or mixed white and red lesions (such as candidiasis, frictional keratosis, or lichen planus). Therefore its clinical diagnosis can be challenging, especially for general dentists or non-oral medicine practitioners (7). A comprehensive evaluation must start by excluding any traumatic or reactive causes, followed by an attempt to gently scrape the lesion to ensure it cannot be removed. If the lesion persists, the adjacent mucosa should be stretched to rule out leukoedema. Once these and other differential diagnoses are excluded, a biopsy must be performed, as histopathological examination is mandatory to provide definitive diagnosis and assess the presence and severity of epithelial dysplasia (mild, moderate or severe) (4).

Even with this systematic diagnostic workflow, the histopathological evaluation of OL has limitations. For example, incisional biopsies of large or multifocal lesions can miss critical dysplastic areas. Histologic similarities with other entities, such as lichen planus, can generate diagnostic doubts (8). Dysplasia grading also remains highly subjective, showing significant inter- and intra-observer variability. With no definitive molecular or immunohistochemical markers to confirm the diagnosis of OL, there is a pressing demand for novel, precise and objective diagnostic approaches (8). In this context, Artificial Intelligence (AI)-driven analysis and decision-support tools hold promise for improving consistency, accuracy, and lesion detection (9).

AI encompasses the theory and creation of computer systems capable of performing tasks with human-like intelligence, including visual perception, speech recognition, decision-making, and language translation (10). Machine Learning (ML) is a subfield of AI that focuses on algorithms that improve their performance through exposure to data. In supervised learning, for example, ML models detect patterns in labeled datasets and apply them to make predictions on new, unseen inputs without explicit programming (11). Deep Learning (DL), on the other hand, extends ML by using computer neural networks (CNN) inspired by the human brain (12). These systems of interconnected nodes or neurons are organized into an input layer, one or more hidden layers, and a final output later. During forward propagation, data passes through these layers to produce an output or prediction, whereas during backpropagation, the neural network is able to adjust its internal weights based on the error between its prediction and the true outcome. This iterative process of propagation and weight adjustment allows the network to self-correct and continually enhance its performance over time (13).

In recent years, numerous studies have investigated the application of ML and DL technologies to support clinicians in diagnosing OL (14); however, a comprehensive synthesis of this evidence is still needed. Therefore, this scoping review aims to systematically synthesize current evidence on the use of artificial intelligence technologies, particularly classification algorithms, for the diagnosis of OL and for the assessment of epithelial dysplasia severity in clinically diagnosed OL lesions.

## Materials and methods

### Protocol, registration and reporting

This scoping review was approved by Ethics and Scientific Committee at the Faculty of Dentistry at Universidad Andres Bello, Viña del Mar, Chile (Acta de Aprobación 08-2024 #PROPRGFO_2024_66). The methodology followed the “Scoping Reviews” chapter of the JBI Manual for Evidence Synthesis (15). The protocol is publicly available on the Open Science Framework (https://osf.io/qgt86/overview), and the final manuscript was prepared in accordance with the PRISMA-ScR checklist (https://www.prisma-statement.org/scoping).

### Research question

This review addressed the question: “How effective are AI-based methods in classifying OL for both clinical and histopathological diagnosis, as well as in assessing and grading the severity of epithelial dysplasia in adult patients across diverse clinical settings?”

### Eligibility criteria

Eligibility was defined using the Population/Concept/Context (PCC) framework: (i) Population: studies involving adult patients diagnosed with OL; (ii) Concept: studies evaluating the diagnostic performance of preliminary ML and/or DL models for OL diagnosis; and (iii) Context: studies conducted in any clinical setting.

Only studies published in the last 25 years in English or Spanish were included. Exclusion criteria were non-primary research (e.g., editorials, letters to the editor, reviews, etc.), studies not addressing the use of ML and/or DL for OL diagnosis, articles lacking clearly defined methods, and unavailability of full-text access.

### Information sources and electronic literature search

Systematic literature searches were conducted in PubMed, SCOPUS, Web of Science, and OVID between March-May 2024, and updated in July 2025. Medical Subject Headings (MeSH) terms were selected based on a pilot search using relevant keywords. The final search queries were:

SCOPUS: “ (“Leukoplakia, oral” OR leukoplakia) AND “artificial Intelligence” AND PUBYEAR > 2,000 AND PUBYEAR < 2025 AND [LIMIT-TO (DOCTYPE, “ar”)] AND [LIMIT-TO (LANGUAGE, “English”) OR LIMIT-TO (LANGUAGE, “Spanish”)] AND [LIMIT-TO (SRCTYPE, “j”)]”.
PubMED: (Leukoplakia, oral OR leukoplakia) AND artificial Intelligence.
Web of Science: ([ALL=(Leukoplakia, oral)] OR ALL=(leukoplakia)) AND ALL=(artificial Intelligence)
OVID: [(Leukoplakia, oral or leukoplakia) and artificial Intelligence].af.

### Study selection

All records were imported into Rayyan (https://www.rayyan.ai/) for reference management. Two reviewers (C.L and T.N) independently screened titles, abstracts, and full texts. Discrepancies were resolved through consultation with C.J and S.N.

### Data extraction and synthesis

Data were extracted using pre-defined Excel templates by reviewers C.L and T.N. Results were synthesized narratively and presented using tables and figures. Critical appraisal of included studies was not performed, as it is not mandatory for scoping reviews (15).

## Results

**Figure 1:**
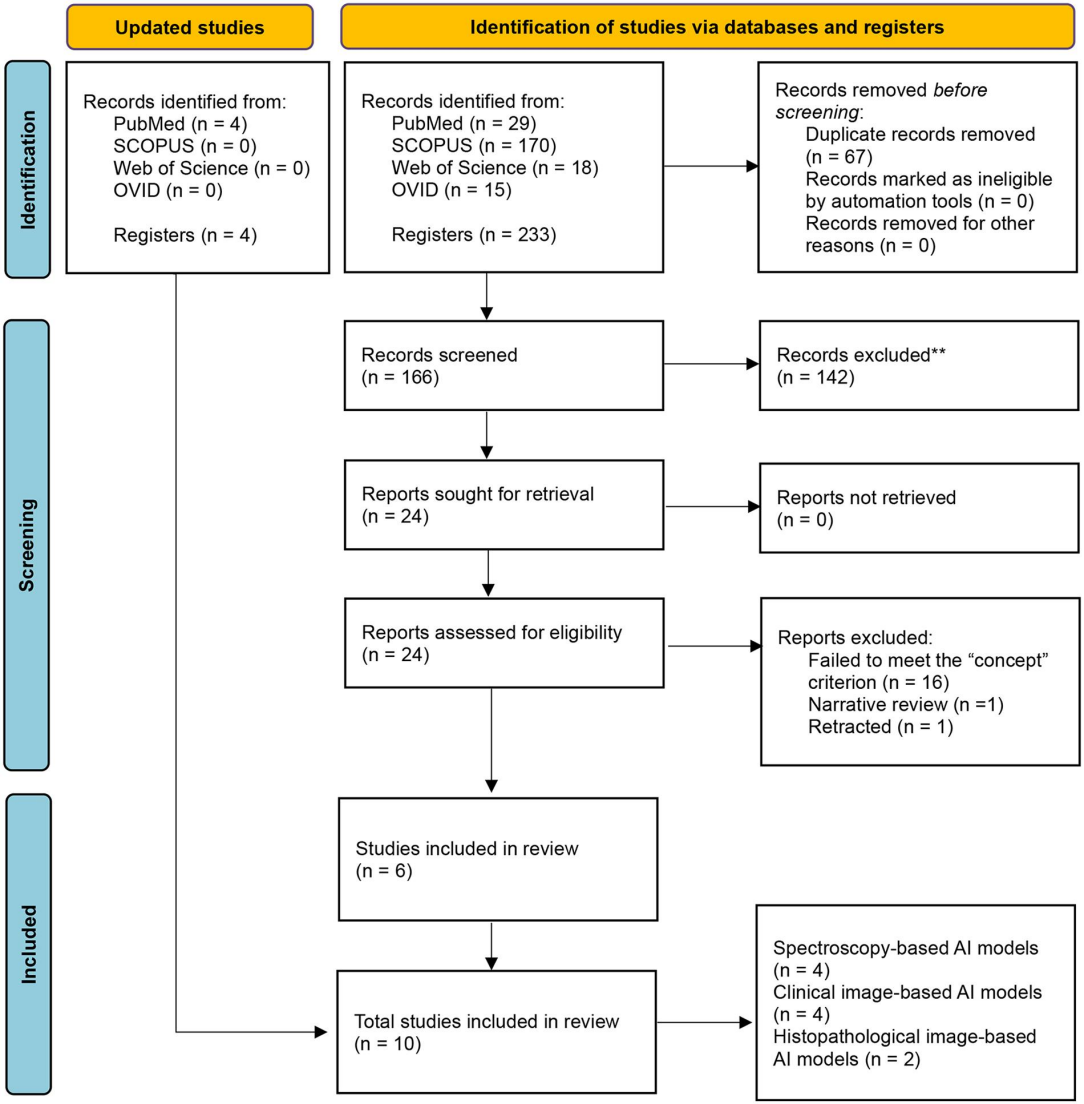
Prisma flow diagram.

The electronic search yielded 233 records. After deduplication removal (*n* = 67), 166 studies were screened based on the predefined eligibility criteria. Of these, 142 were excluded, leaving 24 articles for retrieval and full-text review. Eighteen articles were excluded at this stage for the following reasons: not meeting the “concept” criterion (*n* = 16), being a narrative review (*n* = 1), or having been retracted (*n* = 1). Initially, 6 primary publications were included in this review. However, 4 additional articles were identified during the search update in July 2025, bringing the final total to ten publications. The study selection process is illustrated in the PRISMA-ScR flow diagram (Figure 1).

### Overview of included studies

The current body of literature on AI-based approaches to diagnose OL reflects a dynamic and heterogenous methodological landscape (Table 1). The earliest studies were published in 2000 (16) and 2015 (17), with all subsequent publications published more recently, between 2020 and 2025 (16–25). Main objectives in the field were: (a) OL classification and differentiation from normal oral mucosa (NOM) (16, 23, 25), (b) OL classification and differentiation from OSCC (17–19, 22, 23), (c) OL classification and differentiation from other white lesions of the oral cavity (21, 23, 24), and (d) to assess the severity of OL, particularly through grading of epithelial dysplasia in histological samples (20).

**Table 1:** AI-based diagnostic models for oral leukoplakia.

Ref.	Author (year)	Study design	Objective	Dataset	AI type/model	Task	Main comparison	Key findings
(16)	Van Staveren et al. (2000)	Diagnostic model development study (experimental)	To evaluate the performance of an artificial neural network as an alternative classification technique of autofluorescence spectra of OL, which may reflect the grade of tissue dysplasia.	Autofluorescence spectroscopy measurements	ML/ANN	Classification	OL vs. NOM	The model distinguished abnormal from NOM with 86% sensitivity and 100% specificity and reliably separated homogeneous from non-homogeneous OL. Spectral features showed little to no association with lesion morphology (verrucous vs. erosive) or histologic grade (dysplasia, hyperplasia, hyperkeratosis).
(17)	Banerjee et al. (2015)	Diagnostic model development study (experimental)	Identification of specific label-free biomarkers for differentiation of OL and OSCC.	FTIR spectroscopy measurements	ML/SVM	Classification	OL vs. OSCC	Six spectral features successfully classified OL and OSCC with high sensitivity and specificity. Altered glycogen and keratin content in histological samples could be used to discriminate OL and OSCC.
(25)	Jurczyszyn et al. (2020)	Diagnostic model development study (retrospective)	To propose an effective texture analysis algorithm for OL diagnosis.	Clinical images	ML/PNN	Classification	OL vs. NOM	Differentiation of OL from NOM was highly successful (*p* < 0.05) with the model showing full OL recognition (sensitivity 100%) and specificity 97%.
(18)	Ghosh et al. (2022)	Diagnostic model development study (experimental)	To develop and evaluate a deep reinforced neural network model to classify the epigenetic changes identified from the Raman and FTIR spectra.	FTIR and Raman spectroscopy measurements	DL/DRNN	Classification	OL vs. OSCC	The model achieved an overall accuracy of 83.33% and an ROC of 0.88. Class-specific accuracies for NOM, OL and OSCC were: 83.3%, 87% and 95.24%, respectively.
(20)	Peng et al. (2024)	Diagnostic and grading model development (retrospective)	To establish an objective, accurate and useful detection and grading system for oral epithelial dysplasia in the whole slides of OL	Histopathological images and microarray data	DL/CNN (E-MOD and E-MOD-plus)	Detection + Classification	None (focused on OL dysplasia grading)	E-MOD-plus demonstrated strong internal performance for detecting and grading oral epithelial dysplasia in OL, achieving 81.3% accuracy (95% CI: 71.4–90.5%) with an AUC of 0.793 (95% CI: 0.650 to 0.925). When validated externally on microarray images, accuracy rose to 86.5% (95% CI: 82.4–90.0%) while AUC dipped to 0.669 (95% CI: 0.496 to 0.843). The model outperformed 3 experienced oral pathologists.
(21)	Ramesh et al. (2025)	Diagnostic model development study (retrospective)	To employ and compare the CNNs Xception and MobileNet-v2 for the diagnosis of OL and to differentiate its clinical types from other white lesions of the oral cavity.	Clinical images	DL/CNN (MobileNetV2 and Xception)	Classification	OL vs. other white lesions	Both models were able to diagnose OL and other white lesions using photographs. In terms of F1-score and overall accuracy, the MobilenetV2 model performed noticeably better than Xception (accuracies: 92% and 89%, respectively).
(22)	Schmidl et al. (2025)	Diagnostic model testing (cross-sectional)	To evaluate the application of image recognition by ChatGPT to diagnose OSCC and OL based on clinical images, with images without any lesion as a control group.	Clinical images with or without clinical history.	DL/LLM with vision (ChatGPT 4.0)	Classification	OL vs. OSCC	ChatGPT 4.0 demonstrated the ability to correctly identify OL cases using image recognition alone (sensitivity of 72.2%, specificity of 92.6%, and accuracy of 84.4%), while the ability to diagnose OSCC was insufficient (sensitivity of 18.2%, specificity of 52.2%, and accuracy of 35.6%). However, the diagnostic performance improved by including the clinical history in the prompt (OL performance: sensitivity of 93.3%, specificity of 96,7%, and accuracy of 95,6%; OSCC performance: sensitivity of 100%, specificity of 88.2%, and accuracy of 91.1%). Finally, relying only in clinical history resulted in a misclassification of most OL and some OSCC cases.
(19)	Muniz de Lima et al. (2023)	Diagnostic model development study (retrospective)	To evaluate the importance of complementary data to histopathological image analysis of OL and OSCC for computer-aided diagnosis.	Histopathological images and data	DL/ResNetV2 + Metablock	Multiclass Classification	OL without dysplasia vs. OL with dysplasia vs. OSCC	The highest balanced accuracy for binary classification (OL vs. OSCC) was 95.32%. For multiclass classification (OL without dysplasia vs. OL with dysplasia vs. OSCC), the highest balanced accuracy was 83.24%. The combined use of complementary data and histopathological images achieved a 30.68% gain in performance compared to image-only approaches in multiclass classification tasks.
(23)	Zhang et al. (2025)	Diagnostic model development study (cross-sectional)	To evaluate whether a machine learning model can accurately identify oral mucosal diseases—including OL—based on sub-diffuse reflectance spectroscopy measurements.	Sub-diffuse reflectance spectroscopy measurements.	ML + DL/SVM + PNN	Classification	OL vs. NOM vs. OSCC vs. OLP	Both SVM and PNN yielded comparable accuracy in distinguishing healthy from diseased spectra. Even using only optical parameters features, the models differentiated among OLP, OL, OSCC, and normal mucosa, with high classification metrics, achieving at least 0.8289 accuracy, 0.8495 sensitivity, 0.9311 specificity, and a Matthews correlation coefficient: 0.8085.
(24)	Schwarzler et al. (2025)	Diagnostic model development and validation study (restrospective)	To evaluate whether a deep learning model trained to discriminate 11 classes of oral mucosal lesions could exceed the performance of general dentists.	Clinical images	DL/YOLOv8	Detection + Classification	OL vs. PVL, OLP, Keratosis	For OL, the model achieved a sensitivity of 0.59 and a specificity of 0.94. The F1-score was 0.54 with a precision of 0.5, and an AUCROC 0.86. For PVL, the model achieved a sensitivity of 0.89 and a specificity of 0.97. The F1-score was 0.64 with a precision of 0.5, and an AUCROC 0.91. Keratosis was most likely confused with OL or “white” OLP.In terms of object detection, some of the best results were observed for PVL whereas the lowest values were noted for OL. The overall performance of the final model was comparable to that of oral surgeons (*p* = 0.93), however the model outperformed general dentists (*p* < 0.01),

OL, oral leukoplakia; NOM, normal oral mucosa; OSCC, oral squamous cell carcinoma; PVL, proliferative verrucous leukoplakia; OLP, oral lichen planus; OLP, oral lichen planus; AI, artificial intelligence; FTIR, fourier-transform infrared; AUC, area under the curve; ROC, receiver operating characteristic; DL, deep learning; ML, machine learning; ANN, artificial neural network; SVM, support vector machine; PNN, probabilistic neural network; DRNN, deep reinforced neural network; CNN, convolutional neural network.

A variety of dataset types were utilized, including spectroscopy-based profiles (16–18, 23), clinical and histopathological images (21, 24, 25), as well as multimodal approaches that integrated clinical or histopathological images with other data, such as electronic health records and/or genomic information (19, 20, 22). Research was conducted across multiple countries: India led with 3 publications (17, 18, 21), followed by China with two articles (20, 23) and Germany (22, 24). Single studies originated from Brazil (19), Poland (25), and the Netherlands (16). All articles were published in English, across a diverse array of journals, reflecting the interdisciplinary interest in applying AI models to the diagnosis of OL (Table 1).

### Spectroscopy-based AI models

Early research into AI-based tools for non-invasive diagnosis of OL began in 2000 with the work of Van Staveren et al. (16). The research team explored the use of artificial neural networks to differentiate OL from NOM using tissue autofluorescence measures obtained through spectroscopy. Their model demonstrated promising diagnostic performance, achieving 86% sensitivity and 100% specificity for the task (16). However, it failed to identify specific spectral patterns that consistently reflected on OL morphology or the histopathological grade of dysplasia within lesions (16).

More than a decade later, Banerjee et al. (17) and Ghosh et al. (18) investigated the use of Fourier-transform infrared (FTIR) spectroscopy and combined FTIR/Raman spectroscopy, respectively, to distinguish OL lesions from NOM and OSCC using AI-based algorithms. In Banerjee's study, the best-performing support vector machine (SVM) model, differentiated OL from NOM with 82.1% accuracy, 75% sensitivity, and 91.3% specificity. For distinguishing OL from OSCC, the top-performing model reached 82.1% accuracy, 68.8% sensitivity, and 91.3% specificity. Finally, classification of NOM vs. OSCC achieved 89.7% accuracy, 81.3% sensitivity, and 95.7% specificity (17). In parallel, Gosh's deep reinforced neural network achieved an overall accuracy of 83.33% and an area under the receiver operating characteristic curve (ROC) of 0.88 (18). Notably, class-specific accuracies for identifying NOM, OL, and OSCC were 83.3%, 87%, and 95.24%, respectively (18)— suggesting that model performance improved with increasing lesion severity, a trend consistent with the findings reported by Banerjee et al.

More recently, in 2025, Zhang et al. (23) developed AI models for the detection of OL, OSCC, and oral lichen planus (OLP) through the analysis of sub-diffuse reflectance spectroscopy measurements. Their study demonstrated that both SVM and probabilistic neural network (PNN) models yield comparable performance in distinguishing NOM from abnormal oral mucosa. Furthermore, the models were able to accurately classify OL, OSCC, OLP with high overall accuracy (0.83), sensitivity (0.85) and specificity (0.93).

### Clinical image-based AI models

In addition to spectroscopy-derived features, AI has been increasingly applied to the analysis of clinical images of OL lesions. Jurczyszyn et al. developed a highly effective texture analysis algorithm capable of distinguishing clinical images of OL from NOM with 100% sensitivity and 97% specificity (25).

Another study by Ramesh et al. evaluated two convolutional neural network (CNN) models —MobileNetV2 and Xception—for image-based detection of OL and other common white lesions (21). Although both models achieved strong overall accuracies (92% for MobileNetV2% and 89%for Xception), MobileNetV2 demonstrated consistently higher sensitivity, both for non-homogenous OL (92% vs. 85%) and for other white lesions (94% vs. 91%, respectively). These findings suggest that MobileNetV2 is better suited for identifying more challenging OL lesions (21).

Considering that OL often coexists with or mimics other mucosal conditions, Schwärzler et al. marked a significant milestone in this field by evaluating whether a DL model could discriminate among 11 classes of oral lesions, including OL and proliferative verrucous leukoplakia (PVL) (24). The study also compared the model's diagnostic performance against that of general dentists and oral surgery specialists. For OL, the model achieved moderate performance, with a sensitivity of 0.59, specificity of 0.94, F1-score of 0.54, precision of 0.5, and an AUC-ROC 0.86 (24). In contrast, performance was notably better for PVL, with a sensitivity of 0.89, specificity of 0.97, F1-score of 0.64, precision of 0.5, and AUC-ROC 0.91. Frictional keratosis was frequently misclassified as OL or “white” OLP (24), underscoring the clinical difficulty of visually distinguishing between these lesions. In terms of lesion detection, the model performed better with PVL compared to OL (24). Finally, the model's overall diagnostic performance was comparable to oral surgeon specialists (*p* = 0.93), but significantly outperformed general dentists (*p* = 0.01) (24).

Schmidl et al. introduced a novel diagnostic framework using ChatGPT v4.0 to identify clinical images of OL and OSCC, both with and without accompanying clinical records (22). When relying solely on images, the model demonstrated a reliable performance for OL classification, achieving a sensitivity of 72,2%, specificity of 92,6%, and accuracy of 84,4%. In contrast, its ability to classify OSCC under the same conditions was substantially lower, with a sensitivity of 18,2%, specificity of 52,2%, and accuracy of 35,6%. Interestingly, providing the patients’ clinical records into the prompt greatly improved the model's diagnostic performance across all categories, highlighting the value of integrating visual and contextual data in AI-assisted diagnosis (22). For OL, sensitivity increased to 93.3%, specificity to 96.7%, and accuracy to 95.6%; whereas for OSCC, sensitivity rose to 100%, with specificity of 88.2% and accuracy to 91.1%.

### Histopathological image- and data-based AI models

Histopathological analysis offers a deeper layer of diagnostic information, where AI has also shown considerable promise. In this context, Muniz de Lima et al. developed and evaluated deep CNN algorithms for distinguishing OL from OSCC, using histopathological images supplemented by demographic and clinical data (19). All samples were curated and examined by oral pathologists, who reached a consensus diagnosis for OL and OSCC through microscopic evaluation of biopsy specimens, therefore establishing a gold standard for algorithm training. Among the tested models, the RegNetY fusion with MetaBlock model achieved the highest accuracy (0.952), whereas the PiT and ResNetV2 models achieved the highest sensitivity (0.950) and AUC (0.991), respectively (19).

Another study investigated AI-based approaches for detecting and grading oral epithelial dysplasia (OED) in whole-slide samples of OL (20). The study used both whole-slide images and tissue microarray data to train and validate several CNN models, with all images labeled by expert pathologists serving as the gold standard. Among these, the EfficientNet-B0 model (E-MOD) demonstrated the best performance at the patch level; however, its performance declined at the slide level achieving an overall accuracy of 63.5%, an AUC of 0.673, an average sensitivity of 86.1%, and an average specificity of 64% (20). To improve slide-level interpretability and diagnostic accuracy, the authors developed a two-stage system—named E-MOD-plus—combining 12 feature-specific CNNs to identify histopathological features of dysplasia, followed by a multiclass logistic regression model that integrated these features to predict the overall dysplasia grade. This approach achieved a superior slide-level performance compared to E-MOD alone, with an average accuracy of 86.5%, an average AUC of 0.669, an average sensitivity of 70.6%, and an average specificity of 79.4% (20). Notably, the E-MOD-plus model also outperformed three junior oral pathologists in OED grading accuracy (20), highlighting its potential as an auxiliary tool for both identifying key histopathological features and accurately grading OED severity at the whole-slide level.

## Discussion

OL carries a malignant transformation risk ranging from 1% to 40% (26), underscoring the need for accurate diagnosis and risk stratification, which remains challenging due to its non-specific presentation. Current diagnosis relies heavily on exclusion, combined with a “wait-and-see” approach supplemented by biopsy. However, this method has important limitations, including histological overlap with other oral pathologies and the dependence on the pathologist's expertise, which introduces additional variability and subjectivity into the diagnostic process (27).

Recent advancements in computer sciences and AI-based technologies offer an opportunity to overcome these diagnostic barriers, enhancing the precision and reproducibility of OL diagnosis. Both ML and DL models have demonstrated significant value in dentistry, particularly by improving the detection of caries, periodontal disease, apical periodontitis, and salivary gland diseases, among others (28–30). Overall, their diagnostic performance is able to match, and sometimes even surpass, that of experienced specialists, offering a promising complement to traditional diagnostic workflows (28–30).

This scoping review identified ten studies that explored the application of AI in OL diagnosis, most of which were published within the past five years, reflecting both the growing interest and rapid technological evolution of this field. This momentum is further supported by initiatives like the MimoSA UPLOAD and MimoSA ANNOTATE tools, which provide centralized platform for collecting and labeling oral lesion images from worldwide cohorts, thereby accelerating the development of AI algorithms capable to detect high-risk oral potentially malignant disorders (31). Collectively, the included studies spanned multiple regions, including India, China, Brazil, Germany, Poland, and the Netherlands, illustrating broad interdisciplinary and international engagement in the field. However, underrepresentation of other regions and populations may introduce bias into the current evidence landscape and limit global generalizability of results.

Despite the limited number of eligible investigations included in this work (16–25), the findings consistently support the utility of AI-based tools for OL diagnosis. When differentiating OL from NOM, most models achieved moderate-to-high diagnostic performance, with sensitivity, specificity and accuracy values typically exceeding 80%. Comparable results were observed when distinguishing OL from other mimicking conditions, such as OSCC, PVL and other white lesions. Notably, models tended to perform more robustly when identifying clinically complex or advanced lesions, a pattern observed across both spectroscopy- and image-based studies (18, 24). However, this does not necessarily indicate that AI models inherently struggle to recognize subtle presentations of OL *per se*, but rather limitations in training data diversity, underrepresentation of mild lesions within datasets, or differences in model generalization and pattern recognition capabilities (32, 33). Indeed, advanced disease presentations such as OSCC and PVL often display more distinct anatomical or spectroscopic alterations (34, 35), which AI algorithms can more readily recognize and learn to classify.

To improve performance across the full clinical spectrum of OL, future research should prioritize the inclusion of subtle OL cases during model training and validation. Expanding dataset variability through intentional sampling, multicenter collaborations, and open-access data-sharing initiatives could substantially reduce current biases. Furthermore, applying data augmentation strategies, such as image transformations or contrastive learning with clustering techniques, may help to balance underrepresented categories and enhance model generalizability to less conspicuous lesions (36, 37). Integrating molecular biomarker data, particularly those reflecting early epithelial alterations and subepithelial microenvironmental changes associated with malignant transformation (an aspect still largely overlooked in OL research) (26), could further improve OL detection and support risk stratification in the future. This multimodal strategy makes sense as it mirrors clinical reasoning, where visual, contextual and molecular information collectively inform diagnostic judgements.

Beyond data quantity and diversity, the type of data used also plays a critical role in shaping AI performance. In this review, the included investigations used a variety of data modalities including spectroscopy (16–18, 23), clinical photographs (21, 22, 24, 25) and histopathological images (19, 20). In some cases, these were combined with electronic health records and/or genomic data, which appeared to enhance the model's diagnostic performance (19, 22). For example, Muniz de Lima et al. reported a 30.7% increase in OL identification accuracy when clinical data were integrated with histopathological images, compared with image-only approaches (19). Supporting evidence from other medical fields, points in a similar direction: in dermatology, fusion of dermatoscopic images with clinical data has been shown to outperform unimodal models, especially when using advanced fusion methods such as cross-attention (38). Taken together, these findings highlight multimodal data integration as a promising and likely necessary direction for developing clinically robust AI tools for OL diagnosis in the future.

Diagnostic performance also varied according to the underlying algorithmic architecture. Early spectroscopy-based approaches that relied on traditional ML methods demonstrated promising sensitivity and specificity but often lacked correlation with lesion morphology or histopathological grade (16). Subsequent models combining FTIR and Raman analyses achieved higher class-specific accuracies; however, remained at a proof of concept stage and have yet to progress toward clinical validation (18). In contrast, image- and histopathology-based models employing advanced DL architectures, particularly CNNs and hybrid fusion systems such as E-MOD-Plus frameworks, achieved near-human or even superior diagnostic performance, outperforming general dentists in OL identification and some oral pathologists in epithelial dysplasia grading (20, 24). Nevertheless, to date, only one of these studies has validated its findings in an external population (20), underscoring once more the limited generalizability of current models and the need for further multicenter validation efforts.

Finally, it is important to note that none of the AI systems reviewed have been deployed in clinical settings to date. Most remain in preliminary phases of development, with only one study (20) having conducted external validation. The remaining models rely heavily on internal datasets, limiting their generalizability. This highlights a significant translational gap, making clear that these AI models are not yet suitable for use without expert clinical oversight. Moreover, integrating AI workflows into the diagnosis of OL introduces important ethical and practical challenges that warrant explicit discussion. For instance, high-resolution images may unintentionally expose identifiable facial features raising serious privacy concerns. Algorithmic bias is another concern, as most models have been developed using datasets form specific ethnic populations (primarily Indian, Chinese and German), which may hinder performance across diverse or underserved groups, contributing to diagnostic inequities.

### Limitations

The findings of this work should be interpreted with caution, given the inherent limitations of scoping reviews. Despite a broad research question and comprehensive electronic search, only ten studies met the eligibility criteria, restricting the generalizability of findings across diverse clinical settings. In addition, no formal quality or risk bias assessment was performed, making it difficult to judge the overall reliability and strength of the evidence base. The included studies displayed notable methodological differences that limit comparability. Crucially, the reproducibility of findings is compromised by insufficient reporting of key technical details, such as data preprocessing steps (e.g., image normalization, resizing, or augmentation) and model development strategies (e.g., hyperparameter tuning), etc. Furthermore, reference standards significantly varied across studies, ranging from consensus histopathological diagnosis to clinical impression alone, making it difficult to reliably assess the true diagnostic validity for OL, OSCC, NOM, and PVL. Taken together, these factors highlight the need for greater standardization, transparency, and methodological rigor in future research to strengthen confidence in this emerging field.

## Conclusion

Results from this scoping review underscore the considerable promise of AI-based tools as adjuncts for both the clinical and histopathological diagnosis of OL, with reported sensitivity, specificity and accuracy values frequently exceeding 80%. However, the current body of evidence remains at an early developmental stage, characterized by substantial methodological heterogeneity, small sample sizes, and limited external validation. Crucially, current evidence supports the use of AI only as a decision-support system and does not support its independent clinical use, as these tools must always be interpreted by qualified clinicians. To advance toward clinical translation, future research should prioritize standardized reporting frameworks and the external validation of high-performing algorithms in larger, more diverse, and geographically representative cohorts. Furthermore, efforts to enhance model performance across the full clinical spectrum of OL (particularly in subtle presentations), will be essential to improve diagnosis and timely intervention of OL.
